# Progress in Orthopædics

**Published:** 1898-10-08

**Authors:** 


### Progress in Orthopædics.

**On the Reduction of the Deformity of Pott's Disease.**—The opinions of German surgeons on this question, as expressed at the recent Surgical Congress, are of interest at this time, and are given in abstract.[1] Hoffa, of Wurzburg, pointed out that the method of reduction, as practised by Calot, was not free from danger, since there were 14 fatal cases already published, without enumerating those which had not been placed on record. The deaths are attributable to the anæsthetic, to contusions of the spinal cord, to rupture of the pleura or great vessels, and most frequently to tuberculous meningitis. But in the majority of cases the accidents have occurred in the redressment of old-standing deformity, more or less consolidated. Hoffa is, therefore, of opinion that the existence of this condition is a distinct contra-indication. The second contra-indication is when more than two or three vertebræ are affected, and a third is the presence of abscess; whereas, on the contrary, the presence of paralysis is a distinct indication for the operation. It is quite true that cases have been recorded in which paralysis has followed Calot's operation, but in nearly all cases this paralysis has disappeared subsequently. Hoffa, therefore, looks upon those cases as suitable for redressment in which a reduction of the curve occurs when the patient is placed in the prone position. His own method is to provoke lordosis by suspension, and allow the weight of the body to reduce the curve as much as it will. He then puts on a plaster of Paris corset, and keeps the patient recumbent for such a time as he estimates to be sufficient to cure the caries. It is impossible at the present time to make a definite statement as to the remote results of this method. He had applied it 21 times, and had discovered in two cases abscesses which could not be diagnosed previously. In two cases paralysis had existed before treatment. In one of them the redressment effected a sensible diminution of paralysis, whereas the other in no wise benefited. He would finally insist upon the necessity of preventing the formation of the boss by mobilising the vertebral column after the method of Lorenz.

Lorenz, of Vienna, said that in the active treatment of spondylitis it is clear that we ought not to attempt to redress ankylosed but only those cases of spondylitis in which the vertebral column is flexible. The proceeding of redressment as it is executed by Calot presents many inconveniences, of which the first consisted, in his opinion, of the impossibility of properly regulating the force. One other disadvantage resulted in the lesions associated with decubitus. He had modified the method of Calot, and believed that he could now avoid the inconveniences above mentioned. He had constructed an apparatus which fixed the patient in horizontal suspension by the feet and head. If lordosis so obtained in that position did not suffice to correct the deformity, he compressed very lightly the summit of the projection by means of an elastic pad which had a vertical action. This apparatus permitted him to exercise a very gently and measured pressure. The redressment being obtained, he applied immediately a plaster apparatus, which left naked the sides of the projection. He had employed this method in fourteen cases, without the slightest accident. In four cases he had obtained a notable amelioration of the paralysis which had existed before treatment. As to the ultimate question of osseous fixation in this disease, it appeared to him probable that the lost vertebræ were replaced by osseous substance. Vulpius said the following were modifications which he proposed to apply to the method of Calot: In the first place he would substitute the large number of assistants required in the operation by an apparatus very simple, and combining horizontal suspension in the prone position with the apparatus designed by Lorenz. A dynamometer fitted for that purpose allowed him to measure the force employed. The restitution being obtained, he applied the plaster apparatus, always keeping the patient in the same position. He attached great importance to the fact that the head is enveloped in the plaster. He also suspended the patient with the head directly downwards, and had observed the facility with which that position was endured by the patient. He also thought that narcosis was better borne in that position. Applying the apparatus, one must take fixed points, as the pelvis, the occiput, and the chin. He had also attempted to redress the vertebral column in rachitic infants, but had not obtained good success.

Wüllstein had practised the method of redressment as prescribed by Calot upon the bodies of two subjects who had died with spondylitis. In one of these cases, aged 28 years, the separation of the vertebral bodies measured eight centimetres. The dura mater was ruptured and the spinal cord was exposed. In the second case of a child of six years he found two distinct foci of caries. Redressment was done without any mechanical force. The separation of the vertebral bodies was nevertheless three to four centimetres at one spot and four centimetres at another. There were found two abscesses which had not been suspected during life, and he thought that these existed much more frequently than was generally supposed. One would necessarily ask if such a separation of the vertebra could be bridged over. At present it did not seem possible to answer that question, except, perhaps, in the negative. Rapid and sudden redressment appeared to him to be giving place to quiet redressment, and in stages,

[1] La Semaine Medicale, April 23, 1898.

